# From Actions to Habits: Neuroadaptations Leading to Dependence

**Published:** 2008

**Authors:** Henry H. Yin

**Keywords:** Addiction, alcohol and other drug (AOD) dependence, AOD use behavior, brain, neuroadaptation, cerebral networks, neural pathways, basal ganglia, neural plasticity

## Abstract

Recent work on the role of overlapping cerebral networks in action selection and habit formation has important implications for alcohol addiction research. As reviewed below, (1) these networks, which all involve a group of deep-brain structures called the basal ganglia, are associated with distinct behavioral control processes, such as reward-guided Pavlovian conditional responses, goal-directed instrumental actions, and stimulus-driven habits; (2) different stages of action learning are associated with different networks, which have the ability to change (i.e., plasticity); and (3) exposure to alcohol and other addictive drugs can have profound effects on these networks by influencing the mechanisms underlying neural plasticity.

Addiction is a series of misguided actions. Yet how the brain selects and generates actions has received surprisingly little attention in addiction research. In recent years, considerable progress has been made in identifying the neural circuits responsible for the control of goal-directed actions and habit formation. It is becoming increasingly clear that drugs of abuse can alter these neural pathways. This article discusses the mechanisms underlying reward-guided action selection and their implications for research on alcohol addiction.

## The Organization of Cortico-Basal Ganglia Networks

Understanding how the brain generates actions must begin with a discussion of the cortico-basal ganglia networks.^1^ These networks form a hierarchy for motivated behavior (Swanson 2000; Yin and Knowlton 2005, 2006), which consists of variations on a basic motif, a prototypical network critical for behavioral selection. In this network, glutamatergic (excitatory) projection neurons from the cerebral cortex, a highly layered structure, send axons to the nuclei underneath, commonly known as the basal ganglia, which contain γ-aminobutyric acid (GABA)-ergic (inhibitory) projection neurons. The inhibitory outputs from the basal ganglia, in turn, are directed at downstream structures in the brainstem and in various thalamic nuclei whose projections reenter the cortex.

There is reason to believe that the basal ganglia circuits and their intrinsically generated oscillations are responsible for the generation and selection of behavioral programs; and the variations in patterns of connectivity and in the expression of key proteins like membrane receptors may be tailored for different types of global control processes, as described below (Gerdeman et al. 2003; Yin and Knowlton 2006). A striking feature of such control processes is that they can be measured behaviorally using specific tests.

As recent research has shown, normal mechanisms of learning and memory are usurped by exposure to addictive drugs, so that instead of serving normal biological needs they defect to the purpose of drug seeking (Hyman et al. 2006). There is no consensus, however, on precisely what type of learning process is usurped by addictive substances. Current hypotheses focus on the enhancement of craving, or incentive sensitization (Robinson and Berridge 2003), and on the avoidance of harmful consequences of withdrawal, or allostasis (Le Moal and Koob 2007). These hypotheses largely neglect the central issue of how actions are selected. One reason for this neglect is that the chief behavioral measures in the field (e.g., self-administration and conditioned place preference^2^) lack sufficient analytical power to isolate contributions of distinct neural networks. As discussed below, a major challenge in addiction research is to understand the mechanisms underlying these behavioral control processes and how they are affected by exposure to alcohol and other drugs.

## Three Modes of Behavioral Control

What, then, are these control processes and why are they so important for understanding alcohol addiction? In the study of behavior guided by rewards (i.e., appetitive behavior), researchers are now able to distinguish three major modes of behavioral control with simple experimental tests. These three modes are Pavlovian approach,^3^ goal-directed action, and habit. Although these are rather broad classes of behavioral control with simple operational definitions, they shed considerable light on the integrative functions of the cortico-basal ganglia networks.

Preparatory appetitive Pavlovian behaviors (e.g., approaching location of reward and stimuli that predict reward) and goal-directed instrumental actions are both controlled by the anticipation of the reward. For both, reducing the value of the reward (e.g., by selective satiety, in which the animal is sated on the particular reward offered but not other rewards) or taste aversion induction (in which a particular food is paired with an injection of lithium chloride that results in gastric discomfort) can reduce performance (Colwill and Rescorla 1985; Yin and Knowlton 2002). In both, too, performance is controlled by a predictor of reward and the reward itself. But for Pavlovian approach, the predictor of reward is a stimulus arranged by the experimenter and entirely independent of the animal’s behavior, whereas in instrumental behavior the predictor is the self-generated action by the animal. This distinction is revealed by direct manipulation of the postulated contingencies (e.g., increasing the probability of reward independent of the predictor, be it a particular action in the case of instrumental learning or a stimulus in the case of Pavlovian conditioning) (Hammond 1980; Schwartz and Gamzu 1977). Manipulating the relationship between stimulus and outcome specifically affects Pavlovian behavior, whereas manipulating the action–outcome relationship specifically affects instrumental behavior (Dickinson 1994, 1997; Schwartz and Gamzu 1977).

Habit, a third mode of behavioral control, is not affected by changes in outcome value. Habits persist even if the reward becomes less attractive or if the action is not necessary to earn the reward. Unlike appetitive Pavlovian conditional responses, which are controlled by the stimulus–outcome contingency, all instrumental behaviors initially are goal directed and controlled by the action–outcome contingency. The performance of such actions is exquisitely sensitive not only to its causal efficacy (i.e., by the extent to which the outcome depends on the action) but also to the value of the ensuing consequence (Dickinson 1985; Dickinson and Balleine 1993; Yin and Knowlton 2005, 2006). Under certain conditions, such as extensive training, however, such goal-directed actions are transformed into habits.

As shown by a number of studies in the last two decades, habitual control of instrumental behavior emerges gradually with repeated performance and is relatively unaffected by changes either in outcome value (e.g., devaluation) or in instrumental contingency (Adams 1982; Adams and Dickinson 1981). Thus, once lever pressing for a sucrose reward becomes habitual in this sense, induced taste aversion or unlimited exposure to sucrose prior to a probe test––conducted with the lever extended but without the presentation of a reward––will not reduce the rate of lever pressing compared with controls that did not receive the devaluation treatment.

**Figure 1:**
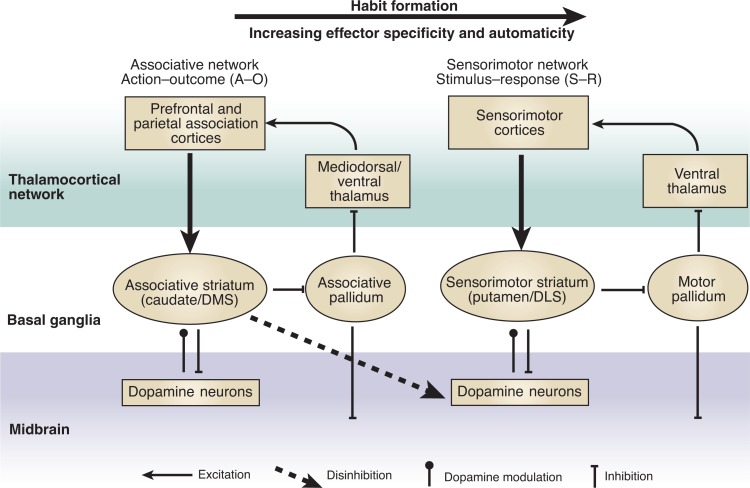
Schematic illustration showing cortico-basal ganglia networks in relation to serial adaptation. A shift from the associative to the sensorimotor cortico-basal ganglia network is observed during habit formation. SOURCE: Yin and Knowlton 2006.

This basic distinction is supported by a series of studies from Yin and colleagues (2004, 2005*a*,*b*, 2006), who established a functional dissociation between associative and sensorimotor striata in the control of instrumental actions. They showed that the associative or medial striatum (similar to most of the caudate nucleus in primates) is critical for the early, goal-directed stage of action learning, whereas the sensorimotor or lateral striatum (similar to the putamen in primates) is more critical for the later, more habitual stage (see figure 1). Together with studies of other structures in these networks (Corbit and Balleine 2003; Corbit et al. 2001, 2002, 2003), this line of research has established that control over instrumental behavior lies with the associative cortico-basal ganglia network in the early stages of learning but switches to the sensorimotor cortico-basal ganglia network in later stages ([Bibr b33-arh-31-4-340][Bibr b34-arh-31-4-340]; Wickens et al. 2007*a*,*b*).

**Figure 2:**
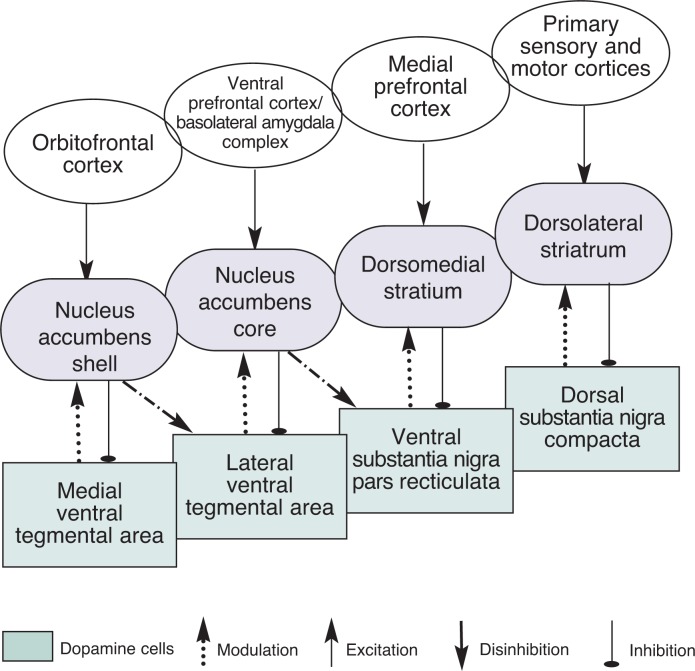
The cortico-basal ganglia networks. An illustration of the major corticostriatal projections and dopaminergic projections in terms of the four major cortico-basal ganglia networks and their corresponding behavioral functions. Emphasis is placed on the spiraling midbrain–striatum–midbrain projections, which allows information to be propagated forward in a hierarchical manner. Note that this is only one possible neural implementation; interactions via different thalamo–cortico–thalamic projections also are possible (Haber 2003). SOURCE: Yin and Balleine 2008.

With respect to the neural adaptations that lead to alcohol dependence, then, the key question is, Which control processes are affected by alcohol as casual drinking becomes compulsive drinking? Drugs of abuse can enhance Pavlovian approach behavior (e.g., approaching environmental stimuli associated with reward), which is largely mediated by the ventral striatum (nucleus accumbens) and the associated cortico-basal ganglia circuit (Corbit et al. 2001; Day et al. 2007; Hyman et al. 2006; Parkinson et al. 2000). In fact, because of the inability to isolate Pavlovian from instrumental modes of behavioral control, current research on addiction has focused almost exclusively on the nucleus accumbens; but we now know that this is only part of the story. As reviewed above, the cortico-basal ganglia networks, which involve the medial (associative) and lateral (sensorimotor) striatal regions above the nucleus accumbens, are responsible for instrumental control processes (see figure 2). Thus, previous work has, by and large, neglected the contributions of the associative and sensorimotor networks in the study of addiction.

## Implications for Alcohol Addiction

A trademark of habitual behavior is that the expected value of the outcome does not affect the behavior. It is as if the value of the outcome has become fixed, so that even if alcohol consumption is associated repeatedly with aversive consequences, such consequences do not alter the performance of the action itself. For this reason, habits have been viewed by some researchers as an intermediate stage before the development of compulsivity (Everitt and Robbins 2005). In the case of alcohol consumption, such a model would emphasize first a shift from casual drinking to habitual drinking, followed by a shift to compulsive drinking. Nonetheless, although the process of habit formation bears a certain resemblance to addiction, addictive behaviors are not the same as enhanced habits (Yin and Knowlton 2005). At first glance, both develop after repeated exposures, and both are insensitive to outcome devaluation. But there are important differences as well. For example, habitual behavior is easily extinguished when the reward is no longer delivered, whereas compulsive behavior is very resistant to extinction (Mowrer 1960). Thus, whereas decades of work has identified the distinct control processes outlined above, we still have little understanding of how these processes interact in producing normal behavior, which rarely is dominated by one process alone. Compulsive behavior, for example, is probably an amalgamation of Pavlovian and instrumental processes.

Appetitive Pavlovian instrumental interactions can take a number of forms. In all, stimuli with incentive value increase the likelihood of action for reward. Although conditioned reinforcement sometimes refers to action-contingent stimuli, Pavlovian instrumental transfer always measures the effect of action-independent stimuli. In conditioned reinforcement, cues produced by instrumental actions can form associations with the reward; and after repeated pairing they become viable reinforcers for the actions (Mowrer 1960). For compulsive drinking, conditioned reinforcement (the feel of the bottle, the taste of alcohol) can play an important role. In Pavlovian instrumental transfer, cues that independently predict reward can elicit central motivational states that enhance instrumental performance. For example, the environmental stimuli associated with drinking (e.g., the sight of a bar) can trigger craving for alcohol and, in turn, alcohol-seeking behavior. Much of the power of advertising, for example, probably derives from the ability of Pavlovian stimuli to trigger motivational states that enhance the selection of certain actions.

The nucleus accumbens is known to play a critical role in Pavlovian instrumental transfer; lesions of this area selectively abolish transfer (Corbit et al. 2001). Interestingly, recent work (Corbit and Janak 2007) has also implicated the dorsal striatum. The sensorimotor striatum in particular appears to play a critical role in the ability of reward-predicting cues to enhance instrumental lever pressing. Such results suggest the possibility of interactions between ventral and more dorsal striatal regions in Pavlovian instrumental interactions.

## The Role of Plasticity

It is possible that all addictive drugs, including alcohol, can affect the capacity for change (i.e., plasticity) in the cortico-basal ganglia networks, thereby altering normal learning processes that are critical for selecting and controlling actions. Although plasticity at all parts of the cortico-basal ganglia network may be involved in addiction, the striatum appears to be the critical node where massive excitatory inputs are transformed into an inhibitory output that ultimately controls behavior (Lo and Wang 2006; Nauta 1989). The glutamatergic transmission can be altered, both presynaptically, in the amount of glutamate released from the axon terminal, and postsynaptically, in the trafficking and expression of various glutamate receptors.

Recent studies (Jedynak et al. 2007; Nelson and Killcross 2006; Porrino et al. 2004) show that exposure to drugs like cocaine and amphetamine can result in significant plasticity in the striatum and potentially accelerate the initial shift from actions to habits. Alcohol may produce similar effects. Acute application of alcohol to brain slices can reverse the direction of plasticity in the associative striatum (Yin et al. 2007). Thus, a train of stimulation that normally leads to increased activity in a striatal region critical for goal-directed actions results in long-term depression instead. One interpretation of these results suggests that the reversal of striatal plasticity could promote habit formation by reducing the overall synaptic strength of the associative striatum, which is a critical component of the brain’s system for the control of goal-directed actions. Previous work (Corbit and Balleine 2003; Corbit et al. 2003; Yin et al. 2004, 2005*a*,*b*, 2006) showed that disrupting the network for goal-directed actions results in a switch to a habitual mode of behavioral control, and vice versa. It remains to be seen if alcohol is able to promote habit formation in vivo by targeting this mechanism.

## Conclusions

The preliminary conceptual framework and the behavioral tests discussed here suggest a number of promising avenues for future study. Researchers can measure, for example, the effects of alcohol on each of these control processes, on their interactions, and on the underlying neural substrates at the cellular level as well as at the level of neural circuits. Further work also can investigate the effects of particular factors (e.g., stress) on susceptibility to addiction and to relapse using the same strategy. The extent of our ignorance in these areas is considerable. An exciting and challenging path lies ahead.

## Footnotes

1 This and other technical terms can be found in the Glossary, pp. 345–347.

2 Conditioned place preference is a commonly used technique to evaluate preferences for environmental stimuli that have been associated with a reward. In general, this procedure involves several trials where the animal is presented with the reward (e.g., food or the effects of a drug of abuse) paired with placement in a distinct environment containing various cues (e.g., tactile, visual, and olfactory). When later tested in the normal state, approaches and the amount of time spent in the compartments previously associated with reward serve as an indicator of preference and a measure of reward learning.

3 In Pavlovian conditioning, a previously neutral stimulus, such as a tone or light, becomes associated with an unconditional stimulus, such as food, to the extent that it will, by itself, evoke a response related to the unconditional response. This new response is called the conditional response.
